# Markers of acute kidney injury in patients with sepsis: the role of soluble thrombomodulin

**DOI:** 10.1186/s13054-017-1815-x

**Published:** 2017-08-25

**Authors:** Shinshu Katayama, Shin Nunomiya, Kansuke Koyama, Masahiko Wada, Toshitaka Koinuma, Yuya Goto, Ken Tonai, Jun Shima

**Affiliations:** Division of Intensive Care, Department of Anesthesiology and Intensive Care Medicine, Jichi Medical University School of Medicine, 3311-1, Yakushiji, Shimotsuke, Tochigi 329-0498 Japan

**Keywords:** Acute kidney injury, Endothelial injury, E-selectin, Sepsis, Soluble thrombomodulin, Plasminogen activator inhibitor-1, Protein C

## Abstract

**Background:**

Endothelial activation and damage occur early during sepsis, with activated coagulopathy and playing a major role in the pathophysiology of sepsis-induced acute kidney injury (AKI). The aim of this study was to compare the various biomarkers of endothelial injury with the biomarkers of coagulation and inflammation and to determine a significant predictor of AKI in patients with sepsis.

**Methods:**

We conducted a single-center, retrospective, observational study on patients with sepsis fulfilling the Third International Consensus Definitions for Sepsis and Septic Shock criteria admitted to an adult intensive care unit (ICU) at a university hospital from June 2011 to December 2016. Levels of 13 biomarkers were measured on ICU admission, including markers of endothelial injury (soluble thrombomodulin [sTM], E-selectin, protein C, and plasminogen activator inhibitor-1 [PAI-1]) and markers of coagulation derangement (platelet count, fibrin degradation product [FDP], prothrombin time [PT], fibrinogen, α_2_-plasminogen inhibitor [α_2_-PI], antithrombin III [AT III], plasminogen, thrombin-antithrombin complex, and plasmin-α_2_-plasmin inhibitor complex). All patients with sepsis were reviewed, and the development of AKI was evaluated. Multivariate logistic regression analysis was performed to identify significant independent predictive factors for AKI.

**Results:**

Of the 514 patients admitted with sepsis, 351 (68.3%) developed AKI. Compared with the non-AKI group, all the endothelial biomarkers were significantly different in the AKI group (sTM [23.6 vs. 15.6 U/ml, *P* < 0.0001], E-selectin [65.5 vs. 46.2 ng/ml, *P* = 0.0497], PAI-1 [180.4 vs. 75.3 ng/ml, *P* = 0.018], and protein C [45.9 vs. 58.7 ng/ml, *P* < 0.0001]). Biomarkers of coagulopathy and inflammation, platelet counts, FDP, PT, α_2_-PI, AT III, plasminogen, and C-reactive protein were significantly different between the two groups. Multivariable logistic regression analysis showed that sTM was an independent predictive factor of AKI, with an AUROC of 0.758 (*P* < 0.0001).

**Conclusions:**

Endothelial biomarkers were significantly changed in the sepsis patients with AKI. Particularly, sTM was an independent predictive biomarker for the development of AKI that outperformed other coagulation and inflammation biomarkers as well as organ function in patients with sepsis.

## Background

Sepsis represents a life-threatening organ dysfunction caused by an aberrant or dysregulated host response to infection [1]. In particular, acute kidney injury (AKI) is one of the most frequent types of organ dysfunction that typically appears early in the course of sepsis. Nearly half of the patients develop AKI in the intensive care unit (ICU) setting, and the presence of AKI in these patients leads to an increase in mortality up to 30–50% [2–4].

The high mortality rate associated with septic AKI may partially be explained by an incomplete understanding of its pathophysiology and a delay in diagnosis. Currently, the diagnosis of AKI is based on either an elevation of serum creatinine levels or the detection of oliguria [5]. These criteria, however, are not specific with respect to the etiology or pathophysiological mechanisms of AKI and have limited sensitivity and specificity for the early recognition of renal dysfunction. The introduction of new biomarkers that are related to the underlying pathogenesis of AKI may allow earlier diagnosis and better prognostication of the clinical course in patients with sepsis.

Endothelial injury is a key feature of sepsis, and the activation and damage of endothelial cells are closely linked with organ dysfunction [6, 7]. Previous studies highlighted systemic hypotension and ischemia-reperfusion injury as the primary pathophysiology involved in septic AKI. Recently, however, it has been shown that septic AKI occurs in the setting of microvascular dysfunction, which is altered by endothelial injury [8–10]. In addition to their role in controlling vasoconstriction/vasodilation and vascular permeability, endothelial cells are essential for maintaining the balance between coagulation and anticoagulation, and they play a prominent role in all three major pathogenic pathways associated with coagulopathy in sepsis: increased tissue factor-mediated thrombin generation; dysfunction of antithrombin and the thrombomodulin-protein C anticoagulant system; and blocked fibrinolysis, which is induced by increased plasminogen activator inhibitor-1 (PAI-1) expression. During sepsis, exposure to inflammatory mediators and interaction with immune cells causes endothelial activation. The activated or injured endothelial cells exert enhanced procoagulatory activities, which contribute to the formation of a large number of microthrombi, leading to microcirculatory failure [6, 11]. Therefore, markers that reflect endothelial injury and its associated coagulopathy could potentially predict the development of AKI and may play a role in promoting clinical research for underlying mechanisms.

To date, several biomarkers of endothelial injury have been evaluated regarding their association with AKI, such as endothelium-derived markers (e.g., thrombomodulin, PAI-1, E-selectin) and markers activated by endothelial cells (e.g., protein C) [12–17]. Most studies, however, were relatively small, conducted in the critical care setting, not focused on sepsis-induced AKI. Also, endothelial function is closely related to coagulopathy; however, there have been few studies in which this relationship has been evaluated. Finally, it remains unclear whether biomarkers of endothelial injury can predict the development of AKI in patients with sepsis.

We report our comparisons of various biomarkers of endothelial injury as predictors of AKI among 514 eligible patients with sepsis admitted to the ICU over a 5-year study period. We also evaluated biomarkers of coagulopathy, parameters of inflammation and organ function, and parameters of endothelial injury to compare their discriminative power in AKI prediction.

## Methods

### Study design and setting

This was a single-center, retrospective, observational study conducted in a 14-bed general ICU of a university hospital (Tochigi, Japan) from June 2011 to December 2016. Laboratory tests, including those for measuring levels of biomarkers of endothelial injury and coagulopathy, were routinely performed at our institute and were partly used for published data [18]. Clinical decisions were made at the discretion of attending ICU physicians. The study protocol was approved by the institutional research ethics committee of Jichi Medical University Hospital. Informed consent was waived because of the retrospective nature of this study.

### Participants

Patients were eligible for enrollment if they were aged 20 years or older and had sepsis at the time of ICU admission. Patients in whom levels of one of the endothelial biomarkers was measured were included in this study. Exclusion criteria included patients with end-stage renal disease requiring dialysis, missing laboratory data on the day of ICU admission, or missing records of hourly urinary output or body weight, which are necessary for the diagnosis of AKI. The prothrombin time (PT) was excluded from the biomarkers of coagulopathy in patients for whom warfarin had been prescribed for any reason. Patient baseline data, including age, sex, body weight, site of infection, and premorbid creatinine level, were collected from electronic medical records. Underlying medical history was also obtained, including ischemic heart disease, chronic heart failure, chronic obstructive pulmonary disease, cerebrovascular accident, diabetes mellitus, or immunocompromised state. Immunocompromised patients were defined as individuals who had been prescribed any immunosuppressive agent. The Acute Physiology and Chronic Health Evaluation II (APACHE II) score [19] and Sequential Organ Failure Assessment (SOFA) score without the renal system parameter (nonrenal SOFA) [20] were used for assessment of illness severity. All patients were reviewed for the development of AKI during the first 48 h of their ICU stay and for the presence of septic shock, overt disseminated intravascular coagulation (DIC), requirement for mechanical ventilation, and mortality at 28 and 90 days.

### Definitions

We defined AKI according to Kidney Disease: Improving Global Outcomes (KDIGO) criteria [5] using an increase in serum creatinine level ≥ 0.3 mg/dl (≥26.5 μmol/L) within 48 h or an increase in serum creatinine ≥ 1.5 times of baseline within 7 days, which corresponds to stage 1 of the KDIGO classification. Chronic kidney disease (CKD) was defined as an estimated glomerular filtration rate (eGFR) < 60 ml/minute/1.73 m^2^ [21] using baseline creatinine. To define the baseline creatinine level, we used a stable value within 1 year before hospital admission as premorbid creatinine. If the baseline creatinine level was not available, the Modification of Diet in Renal Disease equation was used to assume a baseline eGFR of 75 ml/minute/1.73 m^2^ [22]. Sepsis and septic shock were defined according to the Third International Consensus Definitions for Sepsis and Septic Shock [1]. Overt DIC was defined according to the criteria of the International Society on Thrombosis and Haemostasis [23].

### Biomarker measurement and laboratory analysis

Thirteen biomarkers of endothelial injury (soluble thrombomodulin [sTM], PAI-1, protein C, and E-selectin) and coagulation derangement (platelet count, fibrin degradation product [FDP], PT, fibrinogen, α_2_-plasminogen inhibitor [α_2_-PI], antithrombin III [AT III], plasminogen, thrombin-antithrombin complex [TAT], plasmin-α_2_-plasmin inhibitor complex [PIC]) were measured on ICU admission. sTM concentration was determined using the STACIA chemiluminescence enzyme immunoassay with reagent (LSI Medience, Tokyo, Japan). The levels of PAI-1 and E-selectin were measured using tissue plasminogen activator inhibitor and sES latex photometric immunoassays, respectively (Mitsubishi Chemical Medience, Tokyo, Japan). Berichrom® assays (Siemens Healthcare Diagnostics, Tokyo, Japan) were used to determine the activities of protein C, AT III, plasminogen, and α_2_-PI. TAT and PIC F enzyme immunoassays (Sysmex, Hyogo, Japan) were used for measurement of TAT and PIC levels, respectively. Blood coagulation, including those for platelet count, PT, FDP, and fibrinogen, was assayed using the CS-2100i automatic coagulation analyzer (Sysmex). We also measured the following inflammatory and biochemistry parameters on the day of ICU admission: C-reactive protein (CRP), white blood cell count (WBC), blood urea nitrogen (BUN), serum creatinine, albumin, total bilirubin, lactate, brain natriuretic peptide (BNP), and serum cystatin C.

### Statistical analysis

Categorical variables were compared between the two groups using the chi-square test. To determine factors relevant for predicting AKI, we conducted multivariate logistic regression analysis with AKI as the dependent variable and evaluated unadjusted and adjusted ORs. In this analysis, we used model 1 (endothelial biomarkers [sTM and protein C], all coagulation biomarkers including overt DIC, and inflammation parameters [WBC and CRP]) and model 2 (endothelial biomarkers, all coagulation biomarkers including overt DIC, inflammation parameters, organ function [APACHE II, baseline creatinine, BNP, nonrenal SOFA, albumin, total bilirubin, hemoglobin, and presence of septic shock], age, and presence of hypertension) to calculate the adjusted OR. A forward stepwise elimination process was used to remove insignificant variables by each model. The ability of biomarkers to predict AKI was assessed using the AUROC [24]. All analyses were performed using JMP 13 software (SAS Institute, Cary, NC, USA). Data are presented as medians and IQRs (25th–75th percentiles) or percentages. *P* values < 0.05 were considered significant.

## Results

### Enrollment and baseline characteristics

There were 5764 patients considered for participation during the study period. Among these, 651 patients presented with sepsis. Among the patients who were excluded, 50 had received dialysis because of end-stage renal failure, 5 had omission of hourly urinary output or body weight, and 82 had omission of laboratory data on the day of ICU admission. In total, 514 patients were included and divided into the AKI (*n* = 351) and non-AKI (*n* = 163) groups (Table 1). In the AKI group compared with the non-AKI group, age was significantly older (71 vs. 65 years, *P* = 0.003); APACHE II score was significantly higher (26 vs. 19, *P* < 0.0001); and CKD was significantly more common (31.1% vs. 21.5%, *P* = 0.032). Mechanical ventilation was significantly higher (85.8% vs. 76.7%, *P* = 0.011) in the AKI group. The 28-day (15.6% vs. 3.1%, *P* < 0.0001) and 90-day (24.5% vs. 8.4%, *P* < 0.0001) mortality rates were also significantly higher in the AKI group than in the non-AKI group.

**Table 1:** Characteristics of the study population

	All (*n* = 514)	AKI (*n* = 163)	Non-AKI (*n* = 351)	*P* value
Age, years	69 (59–78)	65 (56–74)	71 (61–79)	0.0003
BW, kg	56.7 (48–66)	55 (46–64)	57 (49–67)	0.336
Height, cm	159 (151–166)	159 (152–166)	160 (150–166)	0.823
Male sex	53.9%	53.4%	54.1%	0.873
APACHE II	24 (18–30)	19 (15–25)	26 (20–31)	<0.0001
CKD	28.0%	21.5%	31.1%	0.032
Premorbid creatinine, μmol/L	66.3 (53.0–84.9)	61.9 (47.7–80.0)	70.3 (53.9–89.5)	0.007
Premorbid creatinine, mg/dl	0.75 (0.60–0.96)	0.70 (0.54–0.91)	0.80 (0.0.61–1.01)	0.007
Baseline creatinine, μmol/L	54.8 (50.9–71.6)	55.1 (51.3–65.4)	54.8 (50.8–72.5)	0.011
Baseline creatinine, mg/dl	0.62 (0.58–0.81)	0.62 (0.58–0.74)	0.62 (0.57–0.82)	0.011
Hypertension	48.8%	39.3%	53.3%	0.003
IHD	8.8%	5.5%	10.3%	0.077
CHF	8.8%	4.9%	10.5%	0.036
COPD	5.1%	7.4%	4.0%	0.104
CVA	11.1%	8.6%	12.3%	0.219
DM	25.7%	21.5%	27.6%	0.137
Immunocompromised	29.2%	27.6%	29.9%	0.592
Infection site				0.013
Intracranial	1.2%	2.5%	0.6%	
Head and neck	5.5%	9.2%	3.7%	
Thoracic	22.4%	22.1%	22.5%	
Abdominal	50.8%	53.4%	49.6%	
Soft tissue	6.0%	4.9%	6.6%	
CR-BSI	1.0%	0.0%	1.4%	
UTI	5.3%	3.7%	6.0%	
Others	8.0%	4.3%	10.3%	
Mechanical ventilation	82.9%	76.7%	85.8%	0.011
28-day mortality	11.7%	3.1%	15.6%	<0.0001
90-day mortality	19.3%	8.4%	24.5%	<0.0001

*Abbreviations: AKI* Acute kidney injury, *APACHE II* Acute Physiology and Chronic Health Evaluation II score, *BMI* Body mass index, *BW* Body weight, *CHD* Chronic heart disease, *CKD* Chronic kidney disease, *COPD* Chronic obstructive pulmonary disease, *CR-BSI* Catheter-related bloodstream infection, *CVA* Cerebrovascular accident, *DM* Diabetes mellitus, *IHD* Ischemic heart disease, *UTI* Urinary tract infection

### Laboratory tests and endothelial biomarkers in AKI and non-AKI groups

Table 2 shows the measured variables between the two groups. Among the endothelial biomarkers, sTM (23.6 vs. 15.6 U/ml, *P* < 0.0001) and E-selectin (65.5 vs. 46.2 ng/ml, *P* = 0.0497) levels were both significantly higher in patients with AKI than in those without AKI. Also, protein C activity (45.9% vs. 58.7%, *P* < 0.0001) was significantly lower, and the level of PAI-1 (180.4 vs. 75.3 ng/ml, *P* = 0.018) was significantly higher, in the AKI group. As for parameters of inflammation and organ function, the following were all significantly different in the AKI group compared with in the non-AKI group: CRP, BUN, serum creatinine, cystatin C, BNP, albumin, hemoglobin, lactate, the proportion of patients with septic shock, nonrenal SOFA score, and number of overt DIC cases. As for the coagulation biomarkers, platelet count, PT, FDP, AT III, plasminogen, and α_2_-PI were significantly different for patients in the AKI group compared with in the non-AKI group. In addition, each variable for biomarkers of endothelial injury and coagulopathy was analyzed using AUROC (Table 3). The AUROC values for sTM, E-selectin, protein C, and PAI-1 as predictive factors were 0.758 (0.677–0.825), 0.629 (0.492–0.748), 0.634 (0.581–0.685), and 0.669 (0.566–0.758), respectively.

**Table 2:** Laboratory tests and endothelial biomarkers

	All (*n* = 514)	Non-AKI (*n* = 163)	AKI (*n* = 351)	*P* value
Laboratory tests				
WBC, 10^9^/L	9.9 (4.2–15.0)	9.5 (5.0–14.2)	10.1 (3.7–15.2)	0.255
Hb, g/L	105 (89–121)	107 (93–122)	104 (86–120)	0.023
CRP, mg/L	128 (62–225)	105 (41–186)	137 (70–242)	0.002
Alb, g/L	24 (20–28)	24 (21–28)	23 (19–28)	0.013
T-Bil, μmol/L	15.2 (10.8–24.8)	15.6 (11.1–23.8)	15.0 (10.6–25.8)	0.395
Lactate, mmol/L	2.2 (1.4–3.7)	1.9 (1.2–2.7)	2.5 (1.5–4.6)	<0.0001
BNP, ng/L	136.7 (50.6–395.1)	70.2 (29.8–204.8)	175.0 (68.1–522.7)	0.0003
Renal parameters				
BUN, mmol/L	8.9 (5.4–14.6)	5.4 (3.9–7.9)	11.4 (7.5–16.6)	<0.0001
Creatinine, μmol/L	89.7 (61.0–162.9)	57.5 (47.7–69.0)	127.3 (84.9–206.9)	<0.0001
Creatinine, mg/dl	1.02 (0.69–1.84)	0.65 (0.54–0.78)	1.44 (0.96–2.34)	<0.0001
Cystatin C, mg/L	1.29 (0.91–2.02)	0.86 (0.72–1.03)	1.65 (1.14–2.29)	<0.0001
Coagulation biomarkers				
Platelets, 10^9^/L	144 (91–207)	167 (127–238)	131 (78–193)	<0.0001
FDP, mg/L	16.7 (10.0–27.6)	13.6 (8.9–21.7)	18.9 (10.9–30.5)	0.049
PT, %	56.4 (44.3–70.3)	63.3 (50.3–75.8)	52.4 (42.5–67.6)	<0.0001
Fib, mg/dl	343 (242–487)	373 (261–528)	333 (234–471)	0.076
α_2_-PI, %	75.2 (57.9–97.2)	82.0 (63.3–102.0)	73.6 (56.9–94.0)	0.029
AT III, %	53.7 (41.9–69.0)	60.4 (45.5–76.3)	51.5 (39.3–63.0)	<0.0001
Plasminogen, %	61.4 (46.2–80.3)	66.9 (52.9–88.8)	59.7 (43.6–77.6)	0.0002
TAT, ng/ml	11.1 (6.2–20.0)	9.2 (5.4–16.7)	12.4 (6.7–21.5)	0.088
PIC, μg/ml	1.3 (0.8–2.1)	1.2 (0.8–1.9)	1.3 (0.8–2.2)	0.613
Endothelial activation				
Protein C, %	49.6 (36.3–66.4)	58.7 (44.2–74.0)	45.9 (33.6–63.6)	<0.0001
sTM, U/ml	21.1 (15.4–31.5)	15.6 (12.6–20.5)	23.6 (17.0–38.8)	<0.0001
E-selectin, ng/ml	55.2 (36.2–98.2)	46.2 (32.3–67.5)	65.5 (40.2–131.0)	0.0497
PAI-1, ng/ml	116.3 (53.8–290.8)	75.3 (40.3–150.7)	180.4 (68.0–519.1)	0.018
Other definitions				
No renal SOFA	6 (4–9)	5 (3–7)	7 (5–9)	<0.0001
Overt DIC	24.5%	10.1%	31.6%	<0.0001
Septic shock	45.7%	29.5%	53.3%	<0.0001

*Abbreviations: AKI* Acute kidney injury, *Alb* Albumin, *AT III* Antithrombin III, *BNP* Brain natriuretic peptide, *BUN* Blood urea nitrogen, *CRP* C-reactive protein, *DIC* Disseminated intravascular coagulation, *FDP* Fibrin degradation product, *Hb* Hemoglobin, *PAI-1* Plasminogen activator inhibitor-1, *α*_*2*_ *-PI* α_2_-Plasminogen inhibitor, *PIC* Plasmin-α_2_-plasmin inhibitor complex, *PT* Prothrombin time, *SOFA* Sequential Organ Failure Assessment, *sTM* Soluble thrombomodulin, *TAT* Thrombin-antithrombin complex, *T-Bil* Total bilirubin, *WBC* White blood cell

**Table 3:** AUROCs for predictors of acute kidney injury in sepsis

	AUROC (95% CI)
Coagulation biomarkers	
Platelet	0.627 (0.576–0.675)
FDP	0.614 (0.562–0.663)
PT	0.629 (0.576–0.680)
Fib	0.549 (0.494–0.602)
α_2_-PI	0.564 (0.509–0.618)
AT III	0.618 (0.564–0.670)
Plasminogen	0.600 (0.545–0.652)
TAT	0.591 (0.538–0.642)
PIC	0.527 (0.475–0.579)
Overt DIC	0.607 (0.572–0.641)
Endothelial activation	
Protein C	0.634 (0.581–0.685)
sTM	0.758 (0.677–0.825)
E-selectin	0.629 (0.492–0.748)
PAI-1	0.669 (0.566–0.758)

*Abbreviations: AKI* Acute kidney injury, *AT III* Antithrombin III, *DIC* Disseminated intravascular coagulation, *FDP* Fibrin degradation product, *PAI-1* Plasminogen activator inhibitor-1, *α*_*2*_ *PI* α_2_-Plasminogen inhibitor, *PIC* Plasmin-α_2_-plasmin inhibitor complex, *PT* Prothrombin time, *sTM* Soluble thrombomodulin, *TAT* Thrombin-antithrombin complex

### Multivariate analysis to identify factors predictive of AKI

To identify the predictable factors of AKI, we performed multivariate logistic regression analysis using model 1 and model 2. In both models, sTM was an independent significant predictor of AKI. The nonadjusted OR of sTM was 1.11 (1.06–1.17, *P* < 0.003); the adjusted OR of model 1 was 1.10 (1.04–1.16, *P* = 0.001) and that of model 2 was 1.09 (1.04–1.16, *P* = 0.004) (Table 4).

**Table 4:** Logistic regression analysis for determining predictors of acute kidney injury in sepsis

	OR (95% CI) (unadjusted)	*P*-value	OR (95% CI) (adjusted: model 1)	*P* value	OR (95% CI) (adjusted: model 2)	*P* value
Coagulation biomarkers						
Platelet	1.00 (0.99–1.00)	<0.0001	–	–	–	–
FDP	1.01 (1.00–1.02)	0.022	–	–	–	–
PT	0.98 (0.97–0.99)	<0.0001	–	–	–	–
Fib	1.00 (1.00–1.00)	0.077	–	–	–	–
α_2_-PI	0.99 (0.99–1.00)	0.029	–	–	–	–
AT III	0.98 (0.97–0.99)	<0.0001	–	–	–	–
Plasminogen	0.99 (0.98–0.99)	0.0003	–	–	–	–
TAT	1.02 (1.01–1.04)	0.0004	–	–	–	–
PIC	1.02 (0.95–1.11)	0.603	–	–	–	–
Overt DIC	4.10 (2.39–7.47)	<0.0001	–	–	–	–
Endothelial activation						
Protein C	0.98 (0.98–0.99)	<0.0001	–	–	–	–
sTM	1.11 (1.06–1.17)	<0.0001	1.10 (1.04–1.16)	0.001	1.09 (1.03–1.16)	0.004
E-selectin	1.01 (1.00–1.02)	0.029	–	–	–	–
PAI-1	1.00 (1.00–1.00)	0.007	–	–	–	–

*Abbreviations: AKI* Acute kidney injury, *AT III* Antithrombin III, *DIC* Disseminated intravascular coagulation, *FDP* Fibrin degradation product, *PAI-1* Plasminogen activator inhibitor-1, *α*_*2*_ *PI* α_2_-Plasminogen inhibitor, *PIC* Plasmin-α_2_-plasmin inhibitor complex, *PT* Prothrombin time, *sTM* Soluble thrombomodulin, *TAT* Thrombin-antithrombin complex

Model 1 included endothelial biomarkers, all coagulation biomarkers including overt DIC, and inflammation factors. Model 2 included endothelial biomarkers, all coagulation factors including overt DIC, inflammation factors, organ functions, and age and hypertension

### Relationship of sTM and CKD at time of ICU admission

**Fig. 1:**
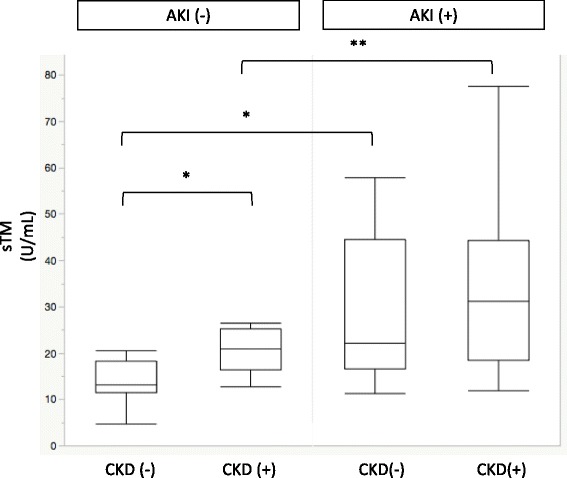
Soluble thrombomodulin (sTM) levels in patients with acute kidney injury (AKI) or without AKI differentiated by the presence of chronic kidney disease (CKD). * *P* < 0.05, ** *P* < 0.01

Researchers in previous studies have reported that sTM was elevated in patients with CKD compared with healthy subjects [24, 25]. Therefore, we analyzed whether the sTM level was different according to the presence or absence of CKD (premorbid eGFR < 60 ml/minute/1.73 m^2^). In the non-AKI group, there was a significant difference in sTM levels between patients who presented with vs. without CKD (20.4 vs. 13.6 U/ml, *P* = 0.049). Furthermore, in both groups with and without CKD, patients who presented with AKI had significantly higher levels of sTM than those without AKI (31.4 vs. 20.4 U/ml, *P* = 0.013; 22.9 vs. 13.6 U/ml, *P* = 0.001, respectively), suggesting that the elevated sTM was related more to the development of AKI than to baseline renal function (Fig. 1).

## Discussion

In this study, the AKI group presented not only endothelial injury but also activation of the coagulation system and the development of organ dysfunction. However, multivariate logistic regression analysis showed that only sTM was an independent significant factor compared with several indices of coagulation activation, inflammation, and organ dysfunction. During the clinical course of sepsis, endothelial injury may occur initially, and then subsequent activation of coagulation leads to organ dysfunction [10]. On the basis of results of multivariable logistic regression analysis, we speculate that endothelial injury occurs as a result of inflammation in sepsis, which in turn results in AKI.

Thrombomodulin (TM) is a thrombin receptor expressed on the surface of endothelial cells. Once thrombin binds with endothelial TM, protein C is activated, and sTM is released into the bloodstream, which inactivates the procoagulant function of thrombin. There is some evidence that increased sTM levels are indicative of endothelial injury, with correlations to DIC, multiple organ failure (MOF), and mortality [7, 26, 27]. However, only three studies have been focused on the relationship between AKI and sTM. In a multicenter, prospective, observational study, sTM and protein C were shown to be predictable markers of AKI in critical care settings [16], whereas in another study, sTM and angiopoietin-2 were shown to play important roles in the development of AKI in patients with acute myocardial infarction [17]. Investigators in another study reported that the high levels of sTM were associated with reduction in the chance of recovery prior to renal function in patients with AKI [28]. However, neither of these studies was focused specifically on patients with sepsis and evaluated the comparison between endothelial activity and coagulopathy. In this study, we used two models of multivariate logistic regression analysis to evaluate the relationship between endothelial function, coagulopathy, and inflammation because these parameters are related to each other. The results indicated that elevated sTM, as a marker of endothelial injury, was a strong independent predictive factor for AKI, regardless of coagulopathy, inflammation, and organ function.

In previous studies, researchers have reported that elevated sTM and protein C levels are strong predictors of AKI in critically ill patients [16]. In this study, protein C was significantly decreased in AKI; however, protein C was not an independent factor in multivariable analysis. This may be partly because our study population comprised patients with sepsis, which is different from previous studies. Coagulation disorders frequently occur in sepsis and are associated with inflammation, which may account for the lack of significant differences in protein C levels between the groups. In this study, we evaluated coagulation function in more detail; however, even when these were factored into the multivariate analysis, only sTM, and not protein C, remained independently predictive of AKI. These results indicate that protein C is more related to coagulation behavior; it may not be a good predictor for AKI.

Some studies have indicated that sTM levels increase in patients with CKD because sTM is excreted by the kidney [25, 29]. Kazama et al. reached this conclusion on the basis of the relationship between decreased urinary TM excretion and increased plasma levels of sTM in patients with CKD [29]. However, no data were available for either creatinine levels or baseline characteristics in their study. In contrast, Lin et al. showed that there was no relationship between sTM and creatinine clearance in patients with septic DIC and/or MOF [7]. Although we did not measure urinary TM in this study, there was a relationship between sTM and CKD in the non-AKI group. However, there was a significant difference in sTM levels in the AKI group, regardless of the presence or absence of CKD. Besides, compared with sTM, the baseline creatinine level was not an independent factor predictive of AKI. In this study, high levels of sTM suggest the predomination of endothelial injury rather than decreases in sTM clearance in patients with sepsis.

In two other studies, researchers found that levels of E-selectin significantly increased in cases of AKI in sepsis, but they measured only the levels of intracellular adhesion molecule-1 (ICAM-1), vascular cell adhesion molecule-1 (VCAM-1), and E-selectin as markers of endothelial injury and did not measure coagulopathy [12, 13]. Therefore, it is unclear whether AKI or coagulation disorders were more predominant, because the presence of coagulation disorders was not verified in these studies. In our study, E-selectin presented with a significantly increased level in the AKI group, but it was not an independent factor with a low AUROC of 0.629. Therefore, we considered it to be insufficient for use as a predictive marker for AKI in sepsis if used as a single parameter.

This study has several strengths. One strength is that this is the first study, to our knowledge, including assessment of sTM as a parameter to evaluate endothelial injury during AKI in sepsis. Another strength is that we provide a detailed examination of coagulation parameters, as well as multivariate analysis and AUROC of the various coagulation parameters, including those of the criteria for overt DIC, which showed that there was an independent relationship between endothelial injury and AKI.

However, our study also has several limitations. First, this study was a single-center, retrospective, observational study. Second, we evaluated four parameters of endothelial injury, but we did not measure other parameters, such as ICAM-1 or VCAM-1. Further studies are needed to evaluate ideal predictive endothelial biomarkers for AKI. Third, data were not available for some biological markers during admission to the ICU. However, this study included a relatively large numbers of patients, which makes these results reliable. Fourth, there was a possibility that AKI had already occurred before admission to the ICU. Therefore, timing of AKI was not always matched for all patients. Further studies are needed to confirm the relationship between endothelial biomarkers and timing of AKI. Finally, we did not evaluate other biomarkers specifically related to AKI, such as urinary neutrophil gelatinase-associated lipocalin [30] and kidney injury molecule-1 [31]. AKI was not only caused by endothelial injury but also confounded by factors such as ischemia or drug toxicity. Therefore, such biomarkers may be superior for the early detection of AKI. However, it is important to evaluate the pathophysiology of AKI in sepsis; it may be associated with any potential clinical intervention for each cause. In this regard, the combination of several biomarkers and definition of AKI may be ideal for evaluating the cause of AKI in sepsis.

## Conclusions

We show that sTM is an independent predictor of AKI in sepsis that is superior to other coagulation and inflammation biomarkers as well as organ function. However, further studies are needed to clarify the relationship between biomarkers of endothelial injury and the development of AKI in sepsis. With time, it is hoped that this will facilitate early recognition of endothelial injury and early treatment.

## Abbreviations

AKI: Acute kidney injury
Alb: Albumin
APACHE II: Acute Physiology and Chronic Health Evaluation II
AT III: Antithrombin III
BMI: Body mass index
BNP: Brain natriuretic peptide
BUN: Blood urea nitrogen
BW: Body weight
CHD: Chronic heart disease
CKD: Chronic kidney disease
COPD: Chronic obstructive pulmonary disease
CR-BSI: Catheter-related bloodstream infection
CRP: C-reactive protein
CVA: Cerebrovascular accident
DIC: Disseminated intravascular coagulation
DM: Diabetes mellitus
eGFR: Estimated glomerular filtration rate
FDP: Fibrin degradation product
Hb: Hemoglobin
ICAM-1: Intracellular adhesion molecule-1
ICU: Intensive care unit
IHD: Ischemic heart disease
KDIGO: Kidney Disease: Improving Global Outcomes
MOF: Multiple organ failure
PAI-1: Plasminogen activator inhibitor-1
α_2_-PI: α_2_-Plasminogen inhibitor
PIC: Plasmin-α_2_-plasmin inhibitor complex
PT: Prothrombin time
SOFA: Sequential Organ Failure Assessment
sTM: Soluble thrombomodulin
TAT: Thrombin-antithrombin complex
T-bil: Total bilirubin
TM: Thrombomodulin
UTI: Urinary tract infection
VCAM-1: Vascular cell adhesion molecule-1
WBC: White blood cell count
